# Women's access to family planning and experiences of reproductive coercion during the COVID-19 lockdown in two districts of Uganda

**DOI:** 10.1016/j.heliyon.2024.e30216

**Published:** 2024-04-25

**Authors:** Mira J. Qureshi, Amanda P. Miller, Stephen Mugamba, Emmanuel Kyasanku, Fred Nalugoda, Robert Malyabe Bulamba, Godfrey Kigozi, Gertrude Nakigozi, James Nkale, Phillip Kato, Grace Kigozi Nalwoga, Stephen Watya, Jennifer A. Wagman

**Affiliations:** a Department of Community Health Sciences, Jonathan and Karin Fielding School of Public Health, University of California, Los Angeles (UCLA), Los Angeles, CA, USA; b Africa Medical and Behavioral Sciences Organization (AMBSO), Plot 7441, Nansana, Hoima Road, Wakiso, P.O Box 37565 Kampala, Uganda; c Division of Epidemiology and Biostatistics, School of Public Health, San Diego State University, San Diego, CA, USA

## Abstract

**Background:**

In March 2020, Uganda enforced country-wide restrictions to control the spread of SARS-CoV-2, categorizing some health services, including family planning (FP), as non-essential. Globally, similar COVID-19 restrictions have been associated with increased vulnerability to reproductive coercion (RC) among women, due to changes in FP service availability and restricted access by partners. This study aims to investigate these dynamics in Uganda, specifically examining the impact of the COVID-19 lockdown on women's access to FP, their experiences of RC, and the relationship between RC and intimate partner violence (IPV).

**Methods:**

We conducted a cross-sectional analysis of data from 960 women participating in the AMBSO Population Health Surveillance Study (APHS) between August 2020 and March 2021 across Wakiso (N = 164) and Hoima (N = 796) districts in Uganda. Our analysis focused on women who were sexually active in the past month, using bivariate analyses to explore the associations between RC and recent experiences of sexual, physical, and verbal IPV.

**Findings:**

The most commonly reported FP methods were injectables (36.8 %) and implants (16 %). Despite the COVID-19 lockdown, less than one percent of participants reported an inability to access their preferred FP method. Notably, 3 % of the women experienced RC in the past 12 months. There was a significant association between RC and sexual IPV (p < 0.0001), as well as physical IPV (p < 0.0001). Instances of verbal IPV were observed to have tripled during the lockdown period.

**Interpretation:**

An increase in verbal IPV was found among women during the COVID-19 lockdown. Additionally, a notable association emerged between other forms of IPV and an increased risk of RC. Despite the lockdown, access to FP remained high, which could be attributed to the prevalent use of long-acting FP methods.

## 1 Introduction

Following Uganda's first confirmed COVID-19 case on March 21, 2020, the government implemented a series of country-wide measures to control infection rates. These included a national curfew, suspension of public transportation, and closure of schools and non-essential businesses, with most restrictions lasting until September 2020 and a full reopening not occurring until January 2022 [1]. While these measures were crucial for slowing the spread of the virus, they inadvertently neglected the broader health and logistical needs of women, impacting access to essential healthcare services, including prenatal care for those who are pregnant, and reliable transportation to healthcare facilities. This oversight not only contributed to increased maternal morbidity and mortality rates [2,3] but also exacerbated challenges in accessing family planning (FP) and other critical health services during this period.

The pandemic's restrictive measures (limiting FP access), combined with financial stress and limited mobility, created an environment with heightened risk for intimate partner violence (IPV) and reproductive coercion (RC) [4], a subset of IPV characterized by behaviors aimed at controlling reproductive health [5]. The World Health Organization defines IPV as any behavior within an intimate relationship, with a current or past partner, that causes physical, psychological, or sexual harm to those in the relationship. IPV can be physical, sexual, emotional or psychological in nature, and also includes control tactics such as RC, which encompasses behaviors that interfere with contraception and pregnancy [[6], [7],^8^], such as pregnancy coercion and contraception sabotage [9]. This heightened vulnerability to IPV and RC during the pandemic has necessitated a deeper exploration of these phenomena.

**Fig. 1:**
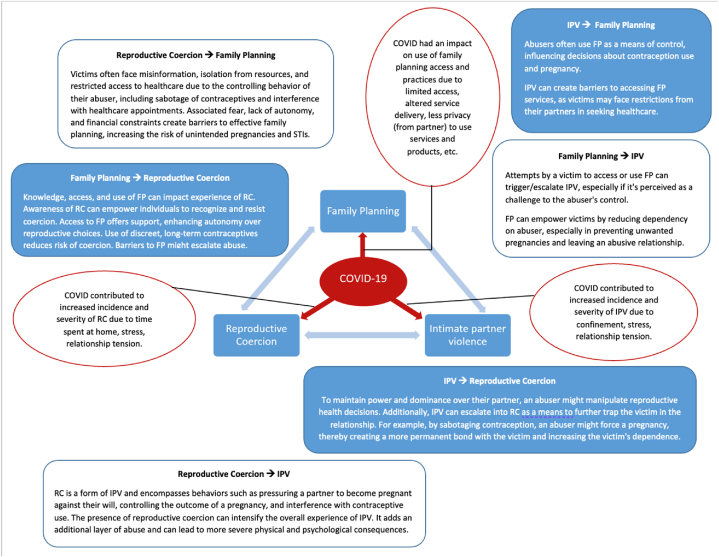
Bidirectional influences of COVID-19 on family planning, reproductive coercion, and intimate partner violence.

We developed a conceptual model (Fig. 1), informed by existing evidence, to explore and better understand how the COVID-19 pandemic intensified the complex bidirectional relationships between IPV, RC, and FP. Drawing on literature that indicates women who experience IPV are at a higher risk of RC [3,10], the model elucidates that IPV can limit access to FP, increasing the likelihood of RC. This relationship is supported by research, such as a study done with women in West Africa that found that nearly half (49.8 %) experienced physical and sexual IPV in their lifetime, with a significant portion (18.6 %) facing RC [11], and a 3.7-fold increase in the likelihood of experiencing RC in the presence of IPV [11]. These relationships are not isolated; IPV is known to have broad-ranging adverse mental, physical, and sexual health effects [[12], [13], [14]]. Moreover, pandemic-induced stressors, such as increased home confinement and relational tension, have amplified IPV and RC [[15], [16], [17]]. Although some studies report consistent IPV rates throughout the pandemic [18], the majority show a significant rise in 10.13039/501100014193IPV incidents during lockdowns [15]. Our model also suggests that increased knowledge and access to FP can empower individuals, potentially mitigating the impact of RC and IPV. This understanding is critical in addressing the research gap on the pandemic's effects on RC and FP [19].

Recognizing the complex relationships between FP, RC and IPV – as we have done in our model - is vital. It reflects the real-world challenges faced during the pandemic, such as Uganda's reclassification of some FP services as non-essential—a decision that, while aimed at resource management, had unintended consequences and inadvertently created access barriers [2,20]. A notable example is an online study highlighting transportation as the major barrier to sexual health clinic access for Ugandan youth, especially the unemployed [21]. This situation, mirrored in regions like South Africa and Ethiopia where FP service utilization declined significantly [22,23], calls for further analysis of pandemic-related barriers to FP services. At the same time, a systematic review of 55 studies across low-income countries found such disruptions were often rare or short-lived [24]. Despite this, the pandemic's global impact on FP was profound, with an estimated drop from 77 % to 71 % in the fulfillment of women's needs for modern contraceptives in 2020, resulting in 60 million fewer users [25]. The World Health Organization underscores the importance of continuous FP access to prevent adverse reproductive health outcomes, especially during global challenges like a pandemic [26].

Given Uganda's position as the nation with the highest global unmet need for FP services (40.6 %) and one of the highest maternal mortality rates in East Africa before the pandemic, there's a pressing need for research to understand the impact of lockdown measures on FP access and women's reproductive autonomy [20,27,28]. Documenting these effects is crucial, not only to address the immediate post-pandemic challenges faced by women in accessing reproductive healthcare but also to inform reproductive health policy and prepare for future health crises [11,29].

To address this critical need, our study examines the ramifications of the COVID-19 lockdown in the districts of Wakiso and Hoima, Uganda, focusing on women's access to preferred FP methods, their experiences of reproductive coercion (RC), and the interplay with recent instances of intimate partner violence (IPV). We hypothesize that the stringent country-wide lockdown measures and additional COVID-19 safety precautions likely exacerbated RC and impeded access to FP. Moreover, social restrictions like curfews and transportation shutdowns might have significantly hindered women's ability to access their chosen FP methods. Further, the study considers the heightened isolation, reduced mobility, and autonomy during the lockdown period, expecting an increase in all forms of RC. In alignment with existing literature that suggests a link between experiences of birth control sabotage and IPV [5,9,10], we also explore the potential correlation between various forms of IPV and RC experiences.

## 2 Methods

### 2.1 Study design and data collection

This study involved an analysis of quantitative survey data collected from September 2020 to March 2021 as part of the second round of the Africa Medical and Behavioral Sciences Organization (AMBSO) Population Health Surveillance (APHS) study. APHS is an ongoing, longitudinal, community population-based, open surveillance cohort conducted in six diverse communities across the Wakiso and Hoima districts of Uganda. The detailed methodology of the APHS study has been previously described [30]. Briefly, the study involves a census-based recruitment strategy, enumerating all households in selected communities and inviting residents aged 13–80 years to participate. The recruitment process includes engagement activities to raise awareness and seek community input. At the data collection hubs, participants undergo an informed consent process, where adults provide written consent and minors give assent. Following consent, they participate in a comprehensive procedure comprising a social and behavioral questionnaire and the collection of biological specimens for HIV and other sexually transmitted infections (STI) testing.

Using a subset of the larger APHS dataset, we specifically analyzed cross-sectional data from 960 non-pregnant, sexually active (i.e., vaginal sex with a partner in the past 30 days) female participants (164 from Wakiso and 796 from Hoima). The second round of APHS, initially started in November 2019, was interrupted by the COVID-19 pandemic lockdown in March 2020 and resumed in August 2020 with an added module to assess the pandemic's impact on FP and RC (see supplementary material for survey module details) that was implemented in four communities - one in Wakiso and three in Hoima. This added component provided the analytical sample for our study. Data collection for the APHS round 2 survey was completed in March 2021.

Our focus centered on women's experiences with FP access, informed by evidence indicating higher rates of IPV and RC among women, as well as adverse outcomes (such as unwanted pregnancy) related to FP access limitations [11,31]. Interviews were conducted in Luganda, Swahili, and Nyoro by trained researchers, adhering to social distancing guidelines and cultural norms, including same-gender interviewer assignments. Data were captured on laptops or paper for subsequent digitization, with continuous staff training on Good Clinical Practice and Human Subjects Protection. Given the sensitive nature of IPV and RC, multiple steps were taken to ensure safe and ethical research conduct, including conducting interviews one-on-one in private spaces, the use of anonymized identifiers and referrals to local support services for participants and staff. Data collection for the APHS round 2 survey was completed in March 2021.

The study received institutional review board (IRB) approval from the Clarke International University - Research Ethics Committee (CIUREC-0059) in Uganda and clearance from the Uganda National Council for Science and Technology (SS 4468). All members of the data collection team received training on how to conduct safe and ethical research on violence, following guidelines developed by the World Health Organization [32].

### 2.2 Measures

#### 2.2.1 Sociodemographic measures

To characterize the study sample, sociodemographic data were analyzed. These included; age (measured as a continuous variable) and marital status which was measured using two questions: “Have you ever been married or entered a consensual union?” (yes, no, don't know) and “Are you currently married?” (yes, no, never married). Community type was a three-level categorical variable: rural, urban, semi-urban. Educational attainment was modeled categorically as no schooling, primary school and secondary school and above. Type of employment was modeled categorically, with multiple response options, including agriculture, housework, government job, student, shopkeeper, trade person or vendor, salon owner, or other.

#### 2.2.2 Biological measures

For biological sample collection, consenting participants provided a 10 ml blood sample, collected by phlebotomists, for HIV and STI testing, done according to the Uganda Ministry of Health guidelines.

#### 2.2.3 Reproductive coercion, family planning and intimate partner violence measures

The RC, FP and IPV measures used in this study were adapted to fit the specific cultural context of Uganda and the objectives of our research. The RC and FP measures were developed by the Evidence-based Measures of Empowerment for Research on Gender Equity (EMERGE) study [33] and both were both adapted from validated tools. For instance, the family planning items came from the Demographic and Health Surveys Program (DHS), which is implemented globally (including in Uganda) and the RC items came from a measure developed in Niger. Both of these modules were adapted from previously validated measures to capture changes in experiences of RC and FP during the COVID-19 lockdown [34,35]. To assess IPV, we utilized a modified version of the Conflict Tactics Scales (CTS) [36], a widely recognized tool for measuring interpersonal conflict, particularly in family and intimate relationships, and frequently used in Uganda [[37], [38], [39], [40]]. The CTS items used in our study were translated and adapted for the APHS research setting by a team member knowledgeable about IPV and proficient in both English and the local languages. These factors ensured the accurate preservation of each item's meaning during translation. The adaptation process included preliminary field testing to evaluate cultural relevance and clarity. We selected items that effectively reflect the key aspects of IPV relevant to our study population, balancing the scale's validity with the practicality of the survey administration.

#### 2.2.4 Reproductive coercion measures

The RC measures consisted of two modules. The first question asked, “In the past year has your husband or male partner done any of the following …” and then provided six questions (standardized Cronbach's alpha = 0.82 for these six items). Participants were asked to indicate yes/no to each question. These questions included “tried to force or pressure you to become pregnant”, “took away your family planning method”, “kept you from going to the clinic or pharmacy to get your family planning method”, “said he would leave you if you didn't get pregnant”, “physically hurt you because you did not become pregnant”, and “he made you feel bad or treated you badly because you did not become pregnant”. A yes response to one or more questions indicated a yes for our overall measure of “experiencing any past year RC”.

The second RC module measured the effect of the pandemic on one's experience of RC and only applied to those who reported experiencing at least one form of RC in the past year in the first question. The second question asked: “If yes, has this happened more, happened less, or not changed since the start of the COVID-19 pandemic and measures to control spread of the virus?” A three-level categorical response variable was given to measure change in experience of each form of RC due to the COVID-19 lockdown: “these things have happened more”, “these things have happened less”, “these things have not changed”.

#### 2.2.5 Family planning measures

A total of five FP measures were used. Current use of a FP method was measured by a yes/no response to the question: “Are you or your partner currently doing something or using any method to delay or avoid getting pregnant?” If the participant responded with yes, they were prompted to answer four additional questions, starting with, “Which method are you currently using to avoid pregnancy?” Thirteen contraceptive methods were listed as options. The next question addressed access to the FP method and asked, “Is your current method also your preferred?” If the participant answered yes, they could skip the next FP question. The following question asked, “What is your preferred method to avoid pregnancy?” and gave the same list of options as the second question. Lastly, the fifth question was asked to all the participants who answered yes to the first question. It measures by a yes/no response to the question “Has the COVID-19 pandemic and the social containment efforts to manage the spread of the virus affected your ability to get your preferred method to avoid pregnancy?” If the participant responded with yes, they were prompted to answer another question with five listed answers stating, “How has it affected your ability to get your preferred family planning method to avoid pregnancy? Participants were able to choose one from five listed options, “The pharmacies and FP counseling clinics are closed”, “Even though the pharmacies and clinics are open, I am not able to get to them due to the social restrictions in place (e.g., curfew, lockdown, no buses) because of the COVID-19 pandemic,” “My family does not allow me to go out because of the COVID-19 pandemic,” “Even though the pharmacies and clinics are open, they do not have my preferred method available,” and “Other reasons (lack of funds, lack of transport, stock outs and too expensive).”

#### 2.2.6 Intimate partner violence measures

A 10-item version of the CTS [36] was used to measure IPV. Experiences of different forms of IPV within the past twelve months (past year) were measured by asking “In the past twelve months has your partner …“: Verbal IPV had one item: “verbally abused or shouted at you?” Physical IPV had six items: “pushed, pulled, slapped, held you down?” “punched you with fist or something that could hurt you?” “kicked or dragged you” “tried to strangle or burn you?” “threatened you with a knife, gun, or other weapon?” and “attacked you with a knife, gun, other weapon?“. Sexual IPV had three items: “used verbal threats to force you to have sex?” “physically forced you to have sex?” and “coerced you to perform other sexual acts when you did not want to?”

### 2.3 Analytic methods

All analyses were conducted using SAS Studio. Initially, the data underwent a thorough quality check for errors, omissions, and outliers. Sociodemographic characteristics were examined, including the analysis of frequencies for dichotomous and categorical variables, as well as measures of central tendency for continuous variables. We specifically focused on the prevalence and changes in experiences of reproductive coercion and access to family planning due to COVID-19, assessing these through frequencies for both dichotomous and categorical variables.

For bivariate analyses, we explored the association between various forms of RC and experiences of verbal, physical, and sexual IPV. This involved using chi-squared (χ2) analysis and Fisher's exact test for variables with small cell counts, supplemented by two-sample T-tests. An alpha level of 0.05 was set to determine statistical significance.

Although we initially considered conducting multivariable logistic regression to adjust for variables that might confound the relationship between IPV and RC experiences, the distribution of our primary variables (IPV and RC) did not support this approach. Consequently, this analysis was not included in our study.

## 3 Results

### 3.1 Sociodemographic and behavioral characteristics

Our analysis included 960 participants, with an average age of 30.6 years (SD 10.2). The majority (72.6 %, n = 696) were currently married, and 12.1 % (n = 116) were previously married. Over half (51.46 %, n = 494) had at least a secondary education. Most participants resided in Hoima (82.9 %, n = 796), with 17.1 % (n = 164) in Wakiso. Urban communities were the most common setting (48.8 %, n = 469), followed by rural areas (32.5 %, n = 313). Notably, 10.9 % (n = 105) were living with HIV. The prevalence of physical and sexual IPV in the past year was 7.6 % (n = 71) and 4.8 % (n = 45), respectively, while verbal IPV was reported by 64.6 % (n = 604) (Table 1).

**Table 1:** Sociodemographic characteristics of analytic sample (n = 960).

Characteristic		n (%)
Age	Mean (SD)	30.6 yrs (10.2 yrs)
Pregnancies	Mean (SD)	4.1 (2.6)
Live Births	Mean (SD)	3.2 (2.2)
Age Categories	13–24	272 (28.3 %)
	25–34	354 (36.9 %)
	35–44	175 (18.2 %)
	45+	159 (16.6 %)
Marital Status (N = 959)		
	Currently Married	696 (72.6 %)
	Previously Married	116 (12.1 %)
	Never Married	147 (15.3 %)
Educational Attainment		
	No Schooling	30 (3.13 %)
	Primary	436 (45.42 %)
	Secondary or above	494 (51.46 %)
Employment	Agriculture	307 (32.0 %)
	Housework	206 (21.5 %)
	Government	43 (4.5 %)
	Student	33 (3.4 %)
	Shopkeeper	56 (5.8 %)
	Trade/Vendor	149 (15.5 %)
	Salon Owner	59 (6.2 %)
	Other	107 (11.2 %)
District		
	Hoima	796 (82.9 %)
	Wakiso	164 (17.1 %)
Community Type		
	Urban	469 (48.9 %)
	Rural	313 (32.5 %)
	Semi-urban	179 (18.6 %)
Verbal IPV (n = 935)		
	Yes	604 (64.6 %)
	No	331 (35.4 %)
Physical IPV (n = 935)		
	Yes	71 (7.6 %)
	No	864 (92.4 %)
Sexual IPV (n = 935)		
	Yes	45 (4.8 %)
	No	890 (95.2 %)

### 3.2 Family planning preferences and pandemic impact

Most participants (56.6 %, n = 543) used FP to avoid pregnancy, with injectables (36.8 %, n = 200) and implants (27.8 %, n = 151) being the most common. Male condoms were used by 10.7 % (n = 58). Notably, 4 % (n = 22) were not using their preferred FP method. Under 1 % (0.9 %, n = 5) reported inability to access their preferred FP method during the lockdown, attributing it to financial constraints, transportation issues, stockouts, and high costs (Table 2).

**Table 2:** Preferred FP method and effect of lockdown on access to preferred FP method.

Characteristic		n (%)^a^
Are you or your partner currently doing something or using any method to delay or avoid getting pregnant? (n = 960)		
	Yes	543 (56.6 %)
	No	417 (43.4 %)
Which method are you currently using to avoid pregnancy (most frequent method in past 30 days) (n = 543)		
	Female sterilization	9 (1.7 %)
	Male sterilization	2 (0.4 %)
	IUD	16 (3.0 %)
	Injectables	200 (36.8 %)
	Implants	151 (27.8 %)
	Oral contraceptive pill	39 (7.2 %)
	Male condom	58 (10.7 %)
	Female condom	0 (0.0 %)
	Emergency contraception	3 (0.6 %)
	Standard days method	27 (5.0 %)
	Lactational amenorrhea method	8 (1.5 %)
	Rhythm method	2 (0.4 %)
	Withdrawal	21 (3.9 %)
	Other method	7 (1.3 %)
Is your current method of family planning, also your preferred method? (n = 543)		
	Yes	521 (96.0 %)
	No	22 (4.0 %)
Has COVID 19 pandemic and the social containment efforts to manage the spread of coronavirus (Lockdown, Curfew, and other social distancing efforts) affected your ability to get your preferred family planning method to avoid pregnancy? (n = 543)		
	Yes	5 (0.9 %)
	No	538 (99.1 %)
How has it affected your ability to get your preferred family planning method to avoid pregnancy? (n = 5)		
	The pharmacies and the family planning counseling clinics are closed	0 (0.0 %)
	Even though the pharmacies and clinics are open, I am not able to get to them due to social restrictions in place (e.g. Curfew, lockdown, no buses) because of the COVID 19 pandemic.	0 (0.0 %)
	My family does not allow me to go out because of the	
COVID-19 pandemic	0 (0.0 %)	
	Even though the pharmacies and clinics are closed, they do not have my preferred method available	1 (20.0 %)
	Other Reasons (lack of funds, lack of transport, stock outs and too expensive)	4 (80.0 %)

a the N for each item varies based on the skip patterns in the survey. See N's reported in rows for total number of participants asked a given question. N (%) that are reported in the column are based on the N for a given item.

### 3.3 Changes in reproductive coercion due to pandemic

Among those reporting RC in the past year, 40.9 % (n = 9) experienced an increase during the lockdown, while 31.8 % (n = 7) saw a decrease and 27.3 % (n = 6) reported no change. Most participants indicated either an increase or no change across all forms of RC, with a minority reporting a decrease (Table 3).

**Table 3:** Changes in Reproductive Coercion Due to Covid-19 Among Women Reporting Reproductive Coercion in the Past 12 months.

Characteristic		n (%) (of those who reported this form of RC in the past year)
Tried to force or pressure you to become pregnant (n = 22/919)		
	More RC	9 (40.9 %)
	Less RC	7 (31.8 %)
	RC unchanged	6 (27.3 %)
Took away your family planning method (n = 5/919)		
	More RC	3 (60.0 %)
	Less RC	1 (20.0 %)
	RC unchanged	1 (20.0 %)
Kept you from going to the clinic or pharmacy to get your family planning method (n = 6/919)		
	More RC	1 (16.7 %)
	Less RC	2 (33.3 %)
	RC unchanged	3 (50.0 %)
Said he will leave you if you didn't get pregnant (n = 15/919)		
	More RC	4 (26.7 %)
	Less RC	3 (20.0 %)
	RC unchanged	8 (53.3 %)
Physically hurt you because you did not become pregnant (n = 3/919)		
	More RC	1 (33.3 %)
	Less RC	1 (33.3 %)
	RC unchanged	1 (33.3 %)
He made you feel bad or treated you badly because you did not get pregnant (n = 8/919)		
	More RC	1 (12.5 %)
	Less RC	1 (12.5 %)
	RC unchanged	6 (75.0 %)

### 3.4 Prevalence of and associations between IPV and RC

Overall, 3.1 % of the sample (n = 28) reported experiencing at least one form of RC in the past year. The most common forms were forcing or pressuring to become pregnant (2.4 %, n = 22), threatening to leave if not pregnant (1.6 %, n = 15), and negative treatment for not getting pregnant (0.9 %, n = 8) (Table 4).

**Table 4:** Bivariate Association Between forms of IPV (Verbal, Physical, and Sexual) and Experiences of Reproductive Coercion Within the Past 12 months.

		Overall (n = 923)*	No Verbal IPV (n = 324)	Verbal IPV (n = 595)	P-value	No Physical IPV (n = 849)	Physical IPV (n = 70)	P-value	No Sexual IPV (n = 874)	Sexual IPV (n = 45)	P-value
Tried to force or pressure you to become pregnant					0.73			0.0004			<0.0001
	yes	22 (2.4 %)	7 (2.2 %)	15 (2.5 %)		16 (1.9 %)	6 (8.6 %)		16 (1.9 %)	6 (13.3 %)	
	no	901 (97.6 %)	317 (97.8 %)	580 (97.5 %)		833 (98.1 %)	64 (91.4 %)		858 (98.2 %)	39 (86.7 %)	
											
Took away your family planning method					1.0			0.33			1.0
	yes	5 (0.5 %)	2 (0.6 %)	3 (0.5 %)		4 (0.5 %)	1 (1.4 %)		5 (0.6 %)	0 (0.0 %)	
	no	918 (99.5 %)	322 (99.4 %)	592 (99.5 %)		845 (99.5 %)	69 (98.6 %)		869 (99.4 %)	45 (100.00 %)	
											
Kept you from going to the clinic or pharmacy to get your family planning method					0.67			0.070			0.26
	yes	6 (0.7 %)	1 (0.3 %)	5 (0.8 %)		4 (0.5 %)	2 (2.9 %)		5 (0.6 %)	1 (2.2 %)	
	no	917 (99.4 %)	323 (99.7 %)	590 (99.2 %)		845 (99.5 %)	68 (97.1 %)		869 (99.4 %)	44 (77.8 %)	
											
Said he will leave you if you didn't get pregnant					0.79			0.00050			0.0046
	yes	15 (1.6 %)	6 (1.9 %)	9 (1.5 %)		9 (1.1 %)	6 (8.6 %)		11 (1.3 %)	4 (8.9 %)	
	no	908 (98.4 %)	318 (98.2 %)	586 (98.5 %)		840 (98.9 %)	64 (91.4 %)		863 (98.7 %)	41 (91.1 %)	
											
Physically hurt you because you did not become pregnant					1.0			0.0163			0.14
	yes	3 (0.3 %)	1 (0.3 %)	2 (0.3 %)		1 (0.1 %)	2 (2.9 %)		2 (0.2 %)	1 (2.2 %)	
	no	920 (99.7 %)	323 (99.7 %)	593 (99.7 %)		848 (99.9 %)	68 (97.1 %)		872 (99.8 %)	44 (97.8 %)	
											
He made you feel bad or treated you badly because you did not get pregnant					0.27			0.0017			0.0053
	yes	8 (0.9 %)	1 (0.3 %)	7 (1.2 %)		4 (0.5 %)	4 (5.7 %)		5 (0.6 %)	3 (6.7 %)	
	no	915 (99.1 %)	323 (99.7 %)	588 (98.8 %)		845 (99.5 %)	66 (94.3 %)		869 (99.4 %)	42 (93.3 %)	
											
At least one form of RC					0.73			<0.0001			<0.0001
	yes	28 (3.1 %)	9 (2.8 %)	19 (3.2 %)		19 (2.2 %)	9 (23.9 %)		20 (2.3 %)	8 (17.8 %)	
	no	895 (97.0 %)	315 (97.2 %)	576 (96.8 %)		830 (97.8 %)	61 (87.1 %)		854 (97.7 %)	37 (82.2 %)	

*Four participants with RC data did not have IPV so N in overall column is greater than N in stratified columns.

Among those who reported verbal IPV in the past year, 3.2 % (n = 19) also reported experiencing at least one form of RC (p = 0.7262). Physical IPV showed a stronger association with RC. Among those who reported physical violence in the past year, 23.9 % (n = 9) experienced at least one form of RC (p < 0.0001). Specifically, 8.6 % (n = 6) reported being forced or pressured to become pregnant (p = 0.0004), and the same percentage reported threats of abandonment for not getting pregnant (p = 0.0005). Additionally, 5.7 % (n = 4) reported negative treatment for not becoming pregnant (p = 0.0017).

The association between sexual IPV and RC was also notable. Of the participants experiencing sexual violence within the last year, 17.8 % (n = 8) reported at least one form of RC (p < 0.0001). Breaking this down further, 13.3 % (n = 6) were forced or pressured into pregnancy (p < 0.0001), 8.9 % (n = 4) faced threats of abandonment for not getting pregnant (p = 0.0046), and 6.7 % (n = 3) experienced negative treatment for the same reason (p = 0.0053).

## 4 Discussion

This study, conducted amidst the ongoing COVID-19 pandemic, provided a critical opportunity to assess the pandemic's impact on family planning services, and experiences of reproductive coercion and intimate partner violence among women in Uganda—a country grappling with high rates of violence against women and challenges in accessing FP and other reproductive health services [9,27,28]. Our findings indicate an increase in IPV, aligning with our hypotheses, and revealing associations between certain forms of IPV and RC. Surprisingly, we found very low rates of pandemic-related issues in accessing FP, which was contrary to our expectations. These insights contribute significantly to the understanding of COVID-19's effects on women's reproductive health and autonomy and are valuable for policy development.

A striking observation was the exceedingly high prevalence of verbal IPV in our sample (64.6 %), a threefold increase from pre-pandemic levels, suggesting that the lockdown conditions likely exacerbated verbal abuse [15]. This pattern is consistent with global trends observed in a UN Women survey conducted across 13 countries, including seven African nations, where verbal IPV emerged as the most prevalent form of violence [[41], [42], [43], [44]].

The significant rise in verbal IPV in our study is a critical factor that policymakers and researchers need to consider when addressing the negative impacts of this and future pandemics. Verbal IPV often serves as a proxy for other forms of IPV, such as physical and sexual violence [45]. Furthermore, the high increase in verbal IPV may be linked to elevated rates of mental distress during the pandemic [46]. It is imperative that national and regional governments enact legislation to assess IPV risk factors, particularly during a pandemic, and develop multi-level solutions to address these issues [41].

While some studies reported unchanged IPV rates during the pandemic [18,47], the majority, including those from the U.S., Uganda, and several other nations, documented an increase in abuse following the onset of the pandemic [9,12,13,15,16]. However, a limitation of our study was the reliance on a single-item measure for verbal IPV, highlighting the need for more comprehensive measures in future research to capture the frequency and severity of IPV, including financial abuse.

The popularity of long-acting reversible contraceptives (LARCs) has been documented in Uganda [28] and is evident in the present study as well, with the majority of participants reporting their use. This trend could indicate a strategic choice during times of service disruption, and LARCs could serve as covert contraceptives against RC. Conversely, a South African study found a preference for provider-independent methods during the pandemic [22]. Both LARCs and provider-independent contraceptives could be recommended to offset lockdown impacts on contraception access. Surprisingly, only a small percentage (0.9 %) of participants in this study reported barriers to accessing their preferred FP method due to the pandemic, with "other" reasons including financial constraints and stockouts.

The unexpectedly low rates of barriers to FP services in our study may be partly attributed to the proximity of healthcare facilities for most participants, reducing their dependence on public transportation. Additionally, the gradual easing of lockdown restrictions during the data collection period could have facilitated easier access to FP services. However, it is crucial to note that our study's sample, though large and encompassing diverse communities, differs from the general population of Uganda, which may limit the generalizability of our findings. This may help explain why contrasting results have been observed in other studies examining FP access during the pandemic across Africa. For instance, an online self-report survey focusing on Ugandan youth revealed that up to 27.2 % were unable to obtain contraceptive supplies during the pandemic. The primary barriers identified included a lack of transportation (68.7 %) and high costs of services (42.2 %), aligning with the main barriers reported in our study [48]. These insights underscore the need for targeted public health interventions in Uganda, particularly initiatives that provide transportation to FP clinics and offer subsidized options for long-lasting FP methods.

In our study, 3 % of participants reported experiencing some form of RC during the pandemic, a figure notably lower than the 8.4 % prevalence reported in the U.S [49]. This disparity might be attributed to underreporting, a common issue when conducting research on sensitive topics like RC. The predominant form of RC observed in our study was coercion into pregnancy, which aligns with the significant association found between sexual partner violence and RC. While a minority of our participants reported a decrease in specific forms of RC, the majority indicated either an increase or no change. This trend suggests that the pandemic might have exacerbated factors contributing to RC, as studies indicate COVID-19 related threats have heightened vulnerabilities to violence [50].

The association of RC with both physical and sexual IPV in our sample is consistent with findings from South Africa and broader sub-Saharan Africa [51,52] and among minoritized women in the U.S [53]. Women experiencing these forms of IPV were more likely to report coercion into pregnancy, raising concerns about increased risks of STIs and unplanned pregnancies. This correlation reinforces the importance of screening for both IPV and RC during family planning visits. This could help identify women at high risk for both forms of violence and highlights the need for targeted interventions to prevent and reduce IPV. Further, interventions that increase access to cost-free contraceptives, STI treatment options, and supportive services to escape abusive relationships are also needed.

Our study is not without limitations. The survey tools, originally designed for a Western context and translated into local languages, may not have fully captured the nuances of the Ugandan context, potentially introducing bias. Recall bias and the cross-sectional nature of the data also limit causal inferences. The small sample size in some categories restricted the depth of our statistical analysis. Furthermore, we must emphasize that while our findings provide valuable insights within our specific sample, generalizing these results to the broader Ugandan population, or the populations of Hoima and Wakiso districts in their entirety, requires caution. The demographic and socio-economic characteristics of our study sample, including its predominantly urban or semi-urban composition and higher levels of education and contraceptive use, differ notably from the general demographic profile of these regions. Therefore, interpretations and policy recommendations based on this study should be considered within the context of these specific sample characteristics.

It is also noteworthy that our adaptation of the Conflict Tactics Scales, though culturally relevant, might have led to underreporting of certain IPV types. Additionally, the lack of pre-COVID baseline data on family planning, reproductive coercion, and intimate partner violence limits our ability to definitively attribute the observed prevalence of these outcomes solely to the impact of the COVID-19 pandemic. Without specific hypothesis testing or comparative pre-pandemic data, we cannot establish a direct causal relationship between the pandemic and the increases in these outcomes. Further, while our study provides valuable insights into the prevalence and impact of physical, sexual, and verbal IPV, we acknowledge the omission of specific measures for controlling behavior and economic dependency, critical aspects of IPV that significantly influence reproductive autonomy. This gap highlights the need for future research to encompass a broader spectrum of IPV dynamics, ensuring a more comprehensive understanding of its multifaceted impact on women's health and decision-making.

Future research should also aim to recruit larger sample sizes for more robust statistical analysis and incorporate more detailed measures of verbal and emotional IPV, including the frequency and severity of violence. Participants may have underreported RC and IPV, affecting the accuracy of our findings. However, measures were taken to ensure privacy and confidentiality during the survey administration to mitigate this risk. Future data collection, including RC and IPV measures specific to the COVID-19 context, will allow for longitudinal analysis to track trends over time and establish more definitive causal relationships. Additionally, qualitative research exploring the impact of IPV on women's access to FP in Uganda is warranted. Our study, conducted across diverse communities, offers findings that are broadly generalizable to Uganda and rural sub-Saharan Africa. Notably, participation rates remained robust despite the pandemic, with a slight increase in the post-lockdown period.

In conclusion, our study highlights the pervasive nature of recent IPV, particularly verbal IPV, and its positive association with RC. The pandemic conditions may have intensified RC among those already affected. Despite these challenges, access to FP remained high, likely due to the prevalence of long-acting contraceptive methods. These findings inform crucial aspects of sexual and reproductive health interventions, emphasizing the need to reduce RC and improve access to preferred contraceptive methods. By addressing these issues, women can experience better health outcomes and have greater control over their fertility and life choices.

## Funding statement

Funding derived from the EMERGE project (10.13039/100000865Bill and Melinda Gates Foundation Grants: OPP1163682 and INV018007; PI Anita Raj). National Institute of Mental Health T32MH080634, and National Institute of Alcoholism and Alcohol Abuse T32AA013525 provided protected time to Amanda Miller to contribute to this manuscript. Mira Qureshi's work was funded in part by NIH T34 GM008563.

## Data availability statement

Data will be made available on request.

## CRediT authorship contribution statement

**Mira J. Qureshi:** Writing – original draft, Methodology, Formal analysis, Conceptualization. **Stephen Mugamba:** Writing – review & editing, Project administration, Methodology, Investigation, Funding acquisition. **Emmanuel Kyasanku:** Writing – review & editing, Resources, Project administration, Methodology, Investigation, Funding acquisition, Data curation, Conceptualization. **Fred Nalugoda:** Writing – review & editing, Supervision, Project administration, Methodology, Investigation, Funding acquisition, Data curation, Conceptualization. **James Nkale:** Writing – review & editing, Supervision, Investigation, Data curation. **Grace Kigozi Nalwoga:** Writing – review & editing, Supervision, Methodology, Investigation, Data curation. **Stephen Watya:** Writing – review & editing, Supervision, Investigation, Funding acquisition, Data curation, Conceptualization.

## Declaration of competing interest

The authors declare that they have no known competing financial interests or personal relationships that could have appeared to influence the work reported in this paper.
