# Supplementary material for: Women's access to family planning and experiences of reproductive coercion during the COVID-19 lockdown in two districts of Uganda
Source: Heliyon. 2024 Apr 25;10(9):e30216. doi: 10.1016/j.heliyon.2024.e30216 (PMC11098776; doi:10.1016/j.heliyon.2024.e30216)
Supplement: Multimedia component 1 [file mmc1.docx]

**QUESTIONNAIRE_ENGLISH**

| **DATA COLLECTOR DETAILS and Date of interview**  Interviewer Number  Date of Interview |  |
| --- | --- |
| **PARTICIPANT IDENTIFIERS**  Participant Unique Identification Number [Insert Unique Identifier]  Participant Physical Location Number  Participant National Identification Number [NIN]  Sex [1=Male; 2-Female]  Names:  Usually called names: _____________________________________  Religious Names: _____________________________________  Other Names: _____________________________________  Mother’s maiden name: _____________________________________ |  |
|  |  |

| Q.1 | How old are you in completed years? **( Valid range 13 years and above)** |  |
| --- | --- | --- |
| Q.2 | When were you born? [Record day, month and year] |  |
| Q.3 | What level of education are you/did you reach? **(Code highest level)**  P1-P4 1  P5-P7 2  S1-S4 3  S5-S6 4  Technical/University 5  Primary professional 6  O-Level professional 7 |  |

| Q.4 | Are you or your partner currently doing something or using any method to delay or avoid getting pregnant?  Yes 1  No 2 |  |
| --- | --- | --- |
| Q.5  Q.6 | Are you or your partner currently using any of the following family planning methods? [*List of FP methods for participants to indicate what applies*]   \|  \| Yes \| No \| DK \| \| --- \| --- \| --- \| --- \| \| Pill \| 1 \| 2 \| 7 \| \| Condom \| 1 \| 2 \| 7 \| \| Spermicide \| 1 \| 2 \| 7 \| \| Self-injection (Sayana) \| 1 \| 2 \| 7 \| \| Injection \| 1 \| 2 \| 7 \| \| Abstinence \| 1 \| 2 \| 7 \| \| Calendar/Rhythm \| 1 \| 2 \| 7 \| \| IUD ( coil) \| 1 \| 2 \| 7 \| \| Breast-feeding \| 1 \| 2 \| 7 \| \| Herbs/traditional medicine \| 1 \| 2 \| 7 \| \| Norplant \| 1 \| 2 \| 7 \| \| Tubal ligation \| 1 \| 2 \| 7 \| \| Withdrawal \| 1 \| 2 \| 7 \| \| Other (specify:__________________) \| 1 \| 2 \| 7 \|   Is your current method of family planning, also your preferred method?  Yes 1  No 2 | \|  \| \| \| --- \| --- \| \|  \| \| \|  \| \| \|  \| \| \|  \| \| \|  \| \| \|  \| \| \|  \| \| \|  \| \| \|  \| \| \|  \| \| \|  \| \| \|  \| \| \|  \| |
| Q.7  Q.8  Q.9 | What are your preferred family planning methods?  **Code up to 3 starting with the most preferred family planning** method    Female Sterilisation 01  Male Sterilisation 02  IUD (coil) 03  Injectables 04  Implants 05  Oral contraceptive pill 06  Male condom 07  Female condom 08  Emergency contraception 09  Standard days method 10  Lactation amenorrhea method 11  Rhythm method 12  Withdrawal method 13  Other methods (specify___________________) 14  None 15  Has COVID 19 pandemic and the social containment efforts to manage the spread of corona virus (Lockdown, Curfew and other social distancing efforts) affected your ability to get your preferred family planning method to avoid pregnancy?  Yes 1  No 2  **If yes**, Ask How has it affected your ability to get your preferred family planning method to avoid pregnancy?  The pharmacies and the family planning  counseling clinics are closed 1  Even though the pharmacies and clinics are open,  I am not able get to them due to social restrictions in place  (e.g. Curfew, lockdown, no buses) because of the COVID 19 pandemic. 2  My family does not allow me to go out because of the  COVID-19 pandemic 3  Even though the pharmacies and clinics are closed,  they do not have my preferred method available 4  others specify _________________________________ 5 |  |
| Q.10 | In the last 12 months, has your husband or male partner done any of the following to you:  (PROMPT)   \|  \| Yes \| No \|  \| \| --- \| --- \| --- \| --- \| \| Tried to force you or pressure you to become pregnant \| 1 \| 2 \| \|___\| \| \| Taken away your family planning method \| 1 \| 2 \| \|___\| \| \| Did not use male condom despite your request \| 1 \| 2 \| \|___\| \| \| Kept you from going to the clinic or pharmacy to get family planning \| 1 \| 2 \| \|___\| \| \| Said he would leave if you didn’t get pregnant \| 1 \| 2 \| \|___\| \| \| Physically hurt you because you did not  become pregnant \| 1 \| 2 \| \|___\| \| \| Made you feel bad or treated you badly  because you did not get pregnant \| 1 \| 2 \| \|___\| \| |  |

| Q.11 | Are you currently married (whether traditional, civil or religious, or in a consensual union)?  Yes 1  No 2 |  |
| --- | --- | --- |

| Q.12 | In the past 12 months, has your sexual partner done any of the following to you? [**PROMPTED] Code as responded to**  Yes No NA(No sexual partner in the past 12  Months)  Verbally abuse or shout at you? 1 2 8  Push, slap or hold you down? 1 2 8  Punch you with fist or with something that could hurt you? 1 2 8  Kick you or drag you? 1 2 8  Tried to strangle or burn you? 1 2 8  Threatened you with a knife, gun, or other weapon? 1 2 8  Attacked you with a knife, gun, or other type of weapon? 1 2 8  Other (specify: ________________________) 1 2 8 |  |
| --- | --- | --- |
| Q.13 | Has your sexual partner ever done any of the following to you? **[PROMPTED]**  Yes No  Used threats to force you to have sex when did not want to? 1 2  Physically forced you to have sex when did not want to? 1 2  Forced you to perform sexual acts when you did not want to do? 1 2 |  |
